# Simultaneous analysis of ^17^O/^16^O, ^18^O/^16^O and ^2^H/^1^H of gypsum hydration water by cavity ring‐down laser spectroscopy

**DOI:** 10.1002/rcm.7312

**Published:** 2015-09-27

**Authors:** Fernando Gázquez, Ian Mather, James Rolfe, Nicholas P. Evans, Daniel Herwartz, Michael Staubwasser, David A. Hodell

**Affiliations:** ^1^ Godwin Laboratory for Palaeoclimate Research, Department of Earth SciencesUniversity of CambridgeDowning StreetCambridgeCB2 3EQUK; ^2^ Institute für Geology und MineralogyUniversität zu KölnGreinstrasse 4‐650939KölnGermany

## Abstract

**Rationale:**

The recent development of cavity ring‐down laser spectroscopy (CRDS) instruments capable of measuring ^17^O‐excess in water has created new opportunities for studying the hydrologic cycle. Here we apply this new method to studying the triple oxygen (^17^O/^16^O, ^18^O/^16^O) and hydrogen (^2^H/^1^H) isotope ratios of gypsum hydration water (GHW), which can provide information about the conditions under which the mineral formed and subsequent post‐depositional interaction with other fluids.

**Methods:**

We developed a semi‐automated procedure for extracting GHW by slowly heating the sample to 400°C *in vacuo* and cryogenically trapping the evolved water. The isotopic composition (δ^17^O, δ^18^O and δ^2^H values) of the GHW is subsequently measured by CRDS. The extraction apparatus allows the dehydration of five samples and one standard simultaneously, thereby increasing the long‐term precision and sample throughput compared with previous methods. The apparatus is also useful for distilling brines prior to isotopic analysis. A direct comparison is made between results of ^17^O‐excess in GHW obtained by CRDS and fluorination followed by isotope ratio mass spectrometry (IRMS) of O_2_.

**Results:**

The long‐term analytical precision of our method of extraction and isotopic analysis of GHW by CRDS is ±0.07‰ for δ^17^O values, ±0.13‰ for δ^18^O values and ±0.49‰ for δ^2^H values (all ±1SD), and ±1.1‰ and ±8 per meg for the deuterium‐excess and ^17^O‐excess, respectively. Accurate measurement of the ^17^O‐excess values of GHW, of both synthetic and natural samples, requires the use of a micro‐combustion module (MCM). This accessory removes contaminants (VOCs, H_2_S, etc.) from the water vapour stream that interfere with the wavelengths used for spectroscopic measurement of water isotopologues. CRDS/MCM and IRMS methods yield similar isotopic results for the analysis of both synthetic and natural gypsum samples within analytical error of the two methods.

**Conclusions:**

We demonstrate that precise and simultaneous isotopic measurements of δ^17^O, δ^18^O and δ^2^H values, and the derived deuterium‐excess and ^17^O‐excess, can be obtained from GHW and brines using a new extraction apparatus and subsequent measurement by CRDS. This method provides new opportunities for the application of water isotope tracers in hydrologic and paleoclimatologic research. © 2015 The Authors. *Rapid Communications in Mass Spectrometry* Published by John Wiley & Sons Ltd.

The isotopic composition of crystallization water of gypsum (CaSO_4_·2H_2_O) has been utilized as a palaeoclimatic proxy to trace geological and hydrogeological processes (see, amongst others,^1^, ^2^, ^3^, ^4^, ^5^, ^6^, ^7^, ^8^). To date, investigations have focused mainly on the analysis and interpretation of the ^18^O and ^2^H values of gypsum hydration water (GHW) and the derived deuterium excess values (d‐excess). This derived parameter is defined as:

$$d‐\text{excess}=\delta^{2}H-8\;\delta^{18}O$$

where δ^2^H and δ^18^O denote ^2^H/^1^H and ^18^O/^16^O in water standardized with respect to V‐SMOW.^9^

Conversely, the relationship between the δ^17^O‐ δ^18^O pair (known as the ^17^O‐excess) in GHW has not been applied yet in hydrology and paleoclimatology. The excess (or depletion) of ^17^O in water is defined as:

$${}^{17}O‐\text{excess}=\ln\left(\delta^{17}O+1\right)-0.528\;\ln\left(\delta^{18}O+1\right)$$

where δ^17^O and δ^18^O denote ^17^O/^16^O and ^18^O/^16^O in water standardized with respect to V‐SMOW.^10^

The addition of the ^17^O‐excess to the isotopic measurements of GHW can provide additional information on the environmental conditions under which the gypsum formed, as well as its post‐depositional interactions with other fluids. For example, the ^17^O‐excess has been shown to be less sensitive to temperature effects than the d‐excess during evaporation.^11^ Thus, combining the ^17^O‐excess and d‐excess recorded by GHW may provide information about the relative effects of humidity and temperature change at the time of gypsum formation.

The most common method for extracting GHW involves step heating a sample in a vacuum line and cryogenically trapping the water.^12^, ^13^ Following extraction, the isotopic composition of the hydration water is determined by isotope ratio mass spectrometry (IRMS, e.g.^14^) or, more recently, by cavity ring‐down laser spectroscopy (CRDS).^6^, ^15^ The latter method allows the simultaneous measurement of oxygen and hydrogen isotopic ratios without the need to convert the water into another gas, thereby minimizing the opportunity for isotopic fractionation.

Here we describe a modification of the extraction procedure introduced by Hodell *et al*.^6^ and report triple oxygen (^16^O, ^17^O and ^18^O) and hydrogen (^1^H and ^2^H) isotope analysis using a new generation CRDS analyzer (Picarro L2140‐*i*).^16^ To validate the ^17^O‐excess values obtained by CRDS, a synthetic gypsum standard and a variety of natural gypsum samples were measured for their δ^17^O and δ^18^O values by fluorination followed by IRMS of O_2_.^17^

## Experimental

### Description of the vacuum assembly

**Figure 1:**
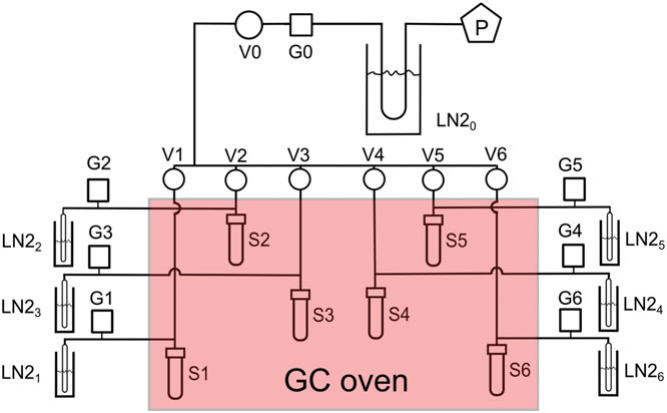
Schematic of the WASP (Water Analyzer Sample Preparation) device developed for the extraction of gypsum hydration water. P: Two‐stage rotation pump; G: Pirani gauges; V: Swagelok® values; S: 12‐mm Pyrex tubes for samples; LN2: Cryogenic traps and 6‐mm OD tubes for freezing GHW.

We developed a semi‐automated procedure for extracting hydration water from gypsum by slowly heating the sample to 400°C *in vacuo* and cryogenically trapping the evolved water. The Water Analyzer Sample Preparation (WASP) system comprises an adapted, programmable gas chromatography (GC) oven and six individual extraction lines attached to a common backing line, coupled to a two‐stage rotary pump (Edwards® E2M2, Crawley, UK) (Fig. 1). A liquid nitrogen (LN2) trap is fitted before the vacuum pump to improve pumping efficiency and avoid backstreaming of pump oil.

Each line is ~1 m in length and composed of ¼‐inch O.D. stainless steel tubing with an I.D. of ~4 mm (Fig. 1). The six lines can be isolated individually by means of Swagelok valves (Swagelok SS‐DSS4, London, UK). Samples are loaded into disposable 12‐mm Pyrex tubes located in the GC oven (ThermoFinnigan TRACE GC; Thermo Scientific, Bremen, Germany) (see Supporting Information). The programmable oven provides complete control over heating steps and rapid cooling following the analysis; this permits several consecutive runs of the apparatus per day. The hydration water is recovered outside the oven in 6‐mm break‐seal tubes by cryogenic trapping in LN2 (Fig. 1). The Pyrex and break‐seal tubes are connected to the stainless steel tubing via ¼‐inch Swagelock Ultra‐torr unions fitted with VitonTM O‐rings, which are replaced after every five or six runs of the apparatus. All the stainless steel lines outside the GC oven are wrapped in heating tape and held at 70°C to prevent condensation of water in the extraction lines.

**Figure 2:**
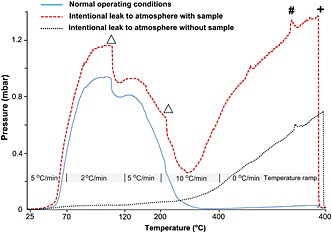
Typical pressure profile recorded during the extraction of GHW. The temperature ramp used along the dehydration process is given. The final pressure differs depending on whether the line is leaking or functioning properly. Vacuum leaks can affect the isotopic composition of the extracted GHW. Δ = raise LN2 trap; # = heat cold spots; + = pump non‐condensable gases.

A Pirani gauge (Edwards® APG100 Active Pirani vacuum gauge) is fitted to each of the six lines for monitoring pressure via an Edwards TIC 6 head instrument controller. The pressure in each line is continuously monitored during the extraction using a computer interface to the WASP system. The change in pressure as a function of time allows identification of any abnormalities (e.g. leaks) during the GHW extraction procedure (Fig. 2).

### Extraction of gypsum hydration water

Gypsum samples (~200 mg for CRDS analyses and ~800 mg for IRMS) were loaded into 12‐mm OD Pyrex tubes and topped with 3 cm of quartz wool to prevent loss of gypsum powder during dehydration. The experiments utilized analytical‐grade powdered gypsum (C/2360/50; Fisher Scientific, Hemel Hempstead, UK) (denoted as NEWGYP standard in this work). In addition, seven natural samples of gypsum were ground to a homogenous powder in an agate mortar and the GHW was extracted using the WASP for subsequent analysis by CRDS and IRMS.

The presence of organic matter and other contaminants in gypsum, including gases derived from microbial activity such as H_2_S and CH_4_ trapped in the mineral lattice, may produce spectral interferences in the CRDS measurements when GHW is analyzed, as has also been reported for the analysis of water extracted from the xylem of plants.^18^, ^19^ Such contamination is likely to be greater in gypsum deposited in environments associated with organic matter degradation, such as lakes and marine settings. To evaluate gypsum formed in a wide variety of depositional environments, our natural sample set included gypsum formed by evaporation in a lake (PI 6A‐13H‐2 8 cm and PI 6C‐7H‐2 26 cm^6^), gypsum derived from seawater evaporation in a coastal setting (Salina 1^8^), gypsum speleothems generated by drip water evaporation in a gypsum cave (SBL‐2.3 and SBL‐8^20^), and two samples of natural gypsum formed in hydrothermal environments (CRI‐01^7^ and BG‐10^21^).

The samples were dried at 45°C for 24 h, and then placed under vacuum to a pressure of ~5 × 10^−3^ mbar for at least 3 h at room temperature to remove adsorbed water. This low‐vacuum pumping (10^−2^–10^−3^ mbar) is effective at removing adsorbed water with no detectable loss of hydration water.^22^ To improve sample throughput, some samples were pumped offline for 3 h in a desiccator, connected to a two‐stage rotary pump (Edwards) and a LN2 trap fitted in‐line between the pump and the desiccator. No difference was found in the isotopic results between the two pumping methods. This allows up to 18 gypsum samples (15 unknowns and 3 standards) to be extracted each day, thereby considerably increasing the sample throughput compared with earlier methods (e.g.^6^).

After the lines had been completely evacuated (pressure ~5 × 10^−3^ mbar), each branch was isolated from the vacuum and the glass 6‐mm break‐seal tubes at the end of each of the lines were immersed in LN2 for water trapping. GHW was released from the samples by slowly heating the sample to 400°C (Fig. 2). The temperature ramp was 5°C/min, between 25°C and 70°C, and reduced to 2°C/min between 70°C and 120°C. In this temperature range gypsum dehydration occurs under low‐to‐medium vacuum conditions.^23^ The slower heating during the second step is required to avoid rapid dehydration of the sample that can lead to gypsum powder blowing through the line. At 110°C, the cryogenic traps were raised (~3 cm) to increase the surface area for freezing the water. The ramp was 5°C/min between 120°C and 200°C. At 200°C, the cryogenic traps were again raised (~5 cm). After this step, the temperature ramp increased to 10°C/min between 200°C and 400°C. Once the oven reached 400°C, this temperature was held constant for 40 min to ensure complete recovery of all water in the lines by the cryogenic traps. Finally, non‐condensable gases were pumped away for 30 s before the 6‐mm break‐seal tubes were flame sealed. The GHW extraction process takes approximately 2 h per run. The pressure in the lines typically reaches 1.2 mbar at 130°C (after 30 min) and then gradually decreases to ~10^−2^ mbar at the end of the heating stage (Fig. 2).

The system was tested for memory effects by extracting the hydration water of isotopically depleted and enriched gypsum in two consecutive runs of the apparatus. These gypsum standards (DPL‐gyp and ENR‐gyp) were produced by hydrating anhydrous CaSO_4_ with isotopically depleted (−8.6‰, −16.1‰ and −131.4‰ for δ^17^O, δ^18^O and δ^2^H values, respectively) and enriched (11.2‰, 21.7‰ and 57.8‰ for δ^17^O, δ^18^O and δ^2^H, respectively) waters, following the procedure of Conley and Bundy.^24^ Prior to the first extraction, the lines were evacuated to a pressure of ~5 × 10^−3^ mbar for 4 days before the first experiments to remove potential adsorbed water remaining from previous samples. The system was evacuated for 30 min between the two consecutive runs.

During GHW extraction, the pressure changes with time can be utilized to identify anomalies caused by vacuum leaks (Fig. 2), water adsorption in the lines, or low amounts of water released by the sample. The system was tested for vacuum leaks that might occur due to deterioration of the Viton O‐rings by intentionally using compromised O‐rings in some of the lines and comparing those results with those from uncompromised O‐rings.

### Distillation of brines

Routine isotopic analysis of brines can produce significant contamination problems in CRDS analyzers due to the accumulation of salts in the valves and pipework and problems with the needle used to inject water into the vaporizer. To solve this issue, we utilized the WASP for the distillation of saline waters prior to CRDS analysis.

Evaporated marine waters from two different brine pools from a natural salt factory (Cabo de Gata Salina, SE Spain, 36°45'32''N, 2°13'08''W) were utilized for the experiments. The water density measured *in situ* using a hydrometer, ranged from 1.13 g cm^−3^ (DEPO‐03) to 1.09 g cm^−3^ (DEPO‐06). Volume of 200 μL of water were loaded into 12‐mm OD tubes and then topped with 3 cm of quartz wool in order to prevent salts from moving through the vacuum line. The samples were loaded into the WASP and a LN2 trap was placed on each sample tube to freeze the water sample and avoid evaporation during vacuum pumping. Subsequently, the assembly was evacuated for 2 min (to a pressure of ~5 × 10^−3^ mbar) and the lines again isolated by closing the Swagelok valves. The LN2 traps were then removed from the sample tubes and placed on the 6‐mm break‐seal tubes. The heating stage was set to a temperature ramp of 5°C/min, with a final temperature of 150°C (maximum pressure ~8 mbar), and held isothermally for 40 min to facilitate complete sample recovery in the traps (final pressure was typically ~2 × 10^−2^ mbar). The traps were raised 5 cm during this final stage. Finally, the lines were evacuated for 30 s (pressure ~5 × 10^−3^ mbar) prior to flame sealing the 6‐mm tubes. In addition to the brines, four internal water standards (JRW, BOTTY, SPIT and ENR) were distilled using the same methodology in order to assess any isotopic fractionation during the extraction procedure. The brines were run in duplicate and analyzed by CRDS, along with the water standards.

### Isotopic measurement of GHW by CRDS

Isotope analyses were conducted at the Godwin Laboratory for Palaeoclimate Research (Department of Earth Sciences) at the University of Cambridge (Cambridge, UK).

The water oxygen and hydrogen isotopes were measured simultaneously by CRDS using a L2140‐*i* Picarro water isotope analyzer for triple oxygen isotope analysis,^16^ interfaced with an A0211 high‐precision vaporizer (both from Picarro, Santa Clara, CA, USA). A Picarro micro‐combustion module (MCM^®^) was used in the majority of the experiments. The MCM comprises an 8‐cm‐long cylindrical cartridge filled with metallic wool coated with a proprietary catalyst, and it was placed in‐line between the vaporizer and the water isotope analyzer. The MCM has been shown to remove combustible compounds from water samples.^18^, ^19^ For experiments that utilized the MCM (denoted as 'MCM On' hereafter) the cartridge was held at a constant temperature of 200°C. For experiments that did not utilize the accessory (denoted as 'MCM Off' hereafter), the WARM mode (~80°C) configuration was used. This configuration avoids water vapour condensation in the MCM transfer line, thereby reducing the memory effect between injections; however, this MCM mode does not remove organics from the vapor stream. Both the MCM On and MCM Off experiments used dry air (containing 21% O_2_) as the carrier gas.

After water extraction, the 6‐mm OD tubes containing 40–60 μL of hydration water were stored until ready for analysis. Prior to analysis, water was frozen into the base of the break‐seal tube by immersion in LN2. Subsequently, the tube was scored with a diamond cutter, broken to a fixed height of 25 mm, and then quickly inserted into the 2‐mL septum‐capped vials used by an A0325 autosampler (Picarro). Each sample was injected ten times into the vaporizer, which was heated to 110°C. Memory effects from previous samples were avoided by rejecting the first three analyses. The values for the final seven injections were averaged with a typical in‐sample precision (±1SD) of ±0.02‰ for δ^17^O values, ±0.04‰ for δ^18^O values, and ±0.19‰ for δ^2^H values, as observed from repeated analysis of an in‐house water standard (SPIT, n = 23) over the period of study.

The results were normalized against V‐SMOW by analyzing internal standards before and after each set of 10 to 12 samples. To this end, four internal water standards (JRW, BOTTY, SPIT and ENR) were calibrated against V‐SMOW and SLAP, using δ^17^O values of 0.0‰ and −29.6986‰, respectively, and δ^18^O values of 0.0‰ and −55.5‰, respectively.^25^, ^26^ All the ^17^O‐excess values are given in per meg units (0.001‰).

The GISP standard water was analyzed during the calibration as an unknown, yielding an average ^17^O‐excess value of 25±12 per meg during the course of the study. This value is in good agreement with the results reported by Schoenemann *et al*.^26^ (22±11 per meg). No measurable differences were observed in the calibration of the liquid water standards when the MCM was utilized. The in‐run drift of the instrument was corrected (when necessary) by the analysis of an internal standard every 3 or 4 samples. The ^17^O‐excess and d‐excess were calculated for each injection using the corrected δ^17^O, δ^18^O and δ^2^H values. The ^17^O‐excess and d‐excess from the seven repeated analyses were then averaged. Typical in‐sample ^17^O‐excess and d‐excess precisions (±1SD) in water standards (SPIT, n = 23) were 13 per meg and 0.2‰, respectively. This agrees with the guaranteed precision given by the manufacturer (15 per meg for ^17^O‐excess). No ^17^O‐excess or d‐excess values were rejected.

### Isotopic measurement of GHW by fluorination‐IRMS method

Fluorination‐IRMS analyses were conducted at the Institute for Geology and Mineralogy at the University of Cologne (Cologne, Germany). An autosampler injects water samples (2.8 μL) into a CoF_3_ reactor held at 375°C which converts H_2_O into O_2_ and HF, following the method developed by Barkan and Luz.^17^ Helium carries the products through a double cold trap immersed in LN2 to freeze out the HF. The O_2_ is then trapped using 5Ǻ molecular sieve held at LN2 temperature. Helium is pumped away after disconnecting the oxygen trap from the He stream and sample O_2_ is transferred to one tube in a 12‐fold sample manifold immersed in LN2. For this study a manifold typically contained four reference water extractions, leaving eight sample tubes for O_2_‐extractions from two or three unknown water samples. To minimize memory effects at least three sample injections were discarded when switching between waters of different isotopic values. The CoF_3_ line is LabView (National Instruments, Austin, TX, USA) controlled and runs fully automated overnight.

The sample O_2_ was then analyzed on a MAT253 isotope ratio mass spectrometer (Thermo Scientific) in dual‐inlet mode at *m/z* 32, 33 and 34. Each measurement comprises 66 samples to reference comparisons divided into 3 blocks of 22 cycles each. Each block begins with a peak centre and adjustment of bellow pressure, such that the ion beam intensity at *m/z* 32 is 8.5 V. The idle time was set to 17 s and the integration time to 12 s. The baseline was measured for 240 s prior to the first sample. The δ^17^O_sample_ and δ^18^O_sample_ values were normalized to V‐SMOW via our internal lab standards that were measured with the samples. As for the CRDS data, SLAP of δ^17^O = −29.6986‰ and δ^18^O = −55.5‰ was used for SMOW‐SLAP scaling.^26^ Analysis of SMOW (n = 15) and SLAP (n = 15) during the course of this study gave −28.699 ± 0.011‰ (1SD) and −53.622 ± 0.024‰ (1SD) for the δ^17^O_measured‐SLAP_ and δ^18^O_measured‐SLAP_ values, respectively. This slightly contracted scale relative to the IAEA‐scale appears to be typical for MAT253 instruments.^26^

## Results and Discussion

### GHW extraction and δ^17^O, δ^18^O and δ^2^H measurements

Our extraction procedure for GHW has several advantages over previous methods, including higher sample throughput and greater precision. The ability to extract five unknowns and a gypsum standard in the same run improves the long‐term reproducibility of the method. In addition, our system allows continuous monitoring of the pressure to detect anomalies in the mineral water extraction procedure.

By monitoring the pressure of the WASP during GHW extraction, vacuum leaks can be detected in individual lines (Fig. 2). When operating correctly, the pressure in the lines displayed similar profiles, first increasing up to 1 mbar at around 120°C, and then decreasing to below 5 × 10^−2^ mbar at the end of the extraction process. These profiles for the lines with compromised O‐rings showed slightly greater pressures (up to 1.2 mbar) at 120°C (Fig. 2). The pressure decreased by as much as 0.2 mbar when the LN2 trap was raised for a second time, but subsequently increased to around 1.4 mbar by the end of the run. In the line run as a blank with no sample, the pressure increased constantly to reach a final value of 0.7 mbar, and no drop in pressure occurred during the run (Fig. 2).

The isotopic composition of the NEWGYP standard extracted in the leaky line showed no difference within error (0.03‰ for the δ^17^O value, 0.03‰ for the δ^18^O value and −51.24‰ for the δ^2^H value) from that obtained from the properly functioning line (0.07‰ for the δ^17^O value, 0.09‰ for the δ^18^O value, and −51.39‰ for the δ^2^H value) (Table 1). However, slight differences were observed in the results of the ENR‐gyp samples. The enriched gypsum extracted in the leaking line showed slightly lower isotope values (12.78‰ for the δ^17^O value, 24.58‰ for the δ^18^O value, and 33.56‰ for the δ^2^H value) than those from the properly functioning line in the same run (12.96‰ for the δ^17^O value, 24.93‰ for the δ^18^O value, and 34.63‰ for the δ^2^H value) (Table 1). This suggests that vacuum leaks can alter the isotopic ratio values of samples extracted using the WASP, especially for hydration waters with an isotopic composition significantly different from that of the ambient water vapour in the laboratory atmosphere. Therefore, it is vital to monitor the pressure in the WASP during GHW extraction to ensure that no leaks occur during extraction.

**Table 1:** Memory effect tests and experiments of vacuum leakage in the WASP lines during GHW extraction

Sample/Line	Line status	δ^17^O (‰)	1σ (‰)	δ^18^O (‰)	1σ (‰)	δ^2^H (‰)	1σ (‰)	^17^O‐excess (per meg)	1σ (per meg)
ENR‐gyp L5	“Fresh” line	12.96	0.04	24.93	0.05	34.73	0.19	−126	9
ENR‐gyp L6	“Fresh” leaking line	12.78	0.04	24.58	0.05	33.56	0.39	−120	8
ENR‐gyp L1	After depleted sample	13.12	0.03	25.24	0.05	35.10	0.38	−125	14
ENR‐gyp L2	After depleted sample	13.08	0.03	25.14	0.05	34.66	0.36	−115	13
NEWGYP‐ L3	“Fresh” line	0.07	0.02	0.09	0.02	−51.39	0.18	20	14
NEWGYP‐L4	“Fresh” leaking line	0.03	0.02	0.03	0.02	−51.24	0.18	17	14
DPL‐gyp L1	“Fresh” line	−6.79	0.04	−12.83	0.06	−149.66	0.24	1	14
DPL‐gyp L2	“Fresh” line	−6.89	0.02	−13.04	0.03	−150.35	0.16	10	9
DPL‐gyp L5	After Enriched sample	−6.75	0.03	−12.77	0.03	−150.75	0.04	11	19
DPL‐gyp L6	After Enriched sample	−6.78	0.02	−12.79	0.02	−150.80	0.11	−10	21

The term “fresh” refers to a line in which the previous sample analyzed was close in isotopic value to the next sample and memory effects should be minimized.

Monitoring the water yield from each sample is also necessary to ensure data quality. The water yield of the sample, measured as the weight loss after the extraction process, can allow incomplete gypsum dehydration and sample contamination (i.e., <100% gypsum) to be detected. The percentage of water in the gypsum samples in our experiments was on average 20.7 ± 0.4 (Table 2). This range is consistent with the stoichiometric percentage of water in gypsum (20.9), which suggests that complete gypsum dehydration took place during the extractions.

**Table 2:** Isotopic analysis (δ^17^O, δ^18^O and δ^2^H values) of hydration water of a gypsum standard (NEWGYP) extracted using the WASP and analyzed by CRDS and IRMS

Line/date	Method	δ^17^O (‰)	1σ (±)	δ^18^O (‰)	1σ (±)	δ^2^H (‰)	1σ (±)	d‐excess (‰)	1σ (±)	^17^O‐excess (per meg)	1σ (per meg)	H_2_O (%)
L3‐28/1/15	CRDS	0.12	0.05	0.21	0.07	−51.23	0.14	−52.3	0.4	21	15	21.3
L4‐29/1/15	CRDS	0.20	0.04	0.35	0.04	−50.83	0.12	−52.8	0.3	27	15	20.8
L5‐30/1/15	CRDS	0.23	0.04	0.42	0.10	−50.91	0.23	−53.3	0.6	19	11	20.9
L4‐30/1/15	IRMS	0.36	0.05	0.67	0.09	‐	‐	‐	‐	7	3	20.5
L5‐30/1/15	IRMS	0.35	0.01	0.65	0.02	‐	‐	‐	‐	10	8	20.6
L2‐30/1/15	IRMS	0.29	0.03	0.52	0.05	‐	‐	‐	‐	11	3	20.9
L1‐30/1/15	IRMS	0.34	0.09	0.62	0.17	‐	‐	‐	‐	10	4	20.7
L2‐30/1/15	IRMS	0.29	0.10	0.53	0.19	‐	‐	‐	‐	9	7	20.6
L3‐30/1/15	IRMS	0.17	0.11	0.32	0.20	‐	‐	‐	‐	0	8	20.8
L4‐30/1/15	IRMS	0.17	0.03	0.31	0.06	‐	‐	‐	‐	10	4	20.8
L5‐30/1/15	IRMS	0.28	0.06	0.51	0.10	‐	‐	‐	‐	9	4	20.4
L6‐30/1/15	CRDS	0.16	0.04	0.25	0.07	−50.89	0.52	−54.2	0.5	18	7	20.9
L1‐03/2/15	CRDS	0.13	0.05	0.16	0.09	−50.34	0.32	−51.6	0.6	33	8	20.8
L2‐03/2/15	CRDS	0.14	0.02	0.25	0.03	−51.28	0.17	−53.3	0.2	8	14	21.9
L4‐04/2/15	CRDS	0.17	0.02	0.30	0.05	−51.48	0.29	−53.9	0.4	20	16	20.4
L5‐05/2/15	CRDS	0.13	0.02	0.21	0.02	−51.81	0.10	−53.5	0.1	23	14	20.3
L1‐09/2/15	CRDS	0.16	0.02	0.27	0.04	−51.59	0.27	−53.8	0.1	13	14	20.7
L2‐09/2/15	CRDS	0.12	0.02	0.18	0.03	−51.88	0.09	−53.3	0.2	21	13	20.5
L3‐10/2/15	CRDS	0.18	0.03	0.29	0.04	−51.63	0.19	−54.0	0.2	18	16	20.9
L6‐18/2/15	CRDS	0.07	0.03	0.11	0.04	−51.80	0.18	−53.4	0.2	20	12	20.8
L1‐22/2/15	CRDS	0.20	0.02	0.33	0.03	−51.24	0.20	−54.7	0.2	32	10	20.7
L3‐04/2/15	CRDS	0.17	0.03	0.30	0.04	−50.39	0.23	−52.9	0.1	11	13	20.7
L4‐02/3/15	CRDS	0.16	0.02	0.29	0.02	−50.80	0.08	−53.1	0.2	12	11	20.1
L1‐03/3/15	CRDS	0.19	0.02	0.32	0.03	−51.27	0.19	−53.8	0.2	17	13	20.9
L2‐05/3/15	CRDS	0.23	0.02	0.41	0.02	−50.36	0.35	−53.5	0.5	18	7	20.6
L3‐06/3/15	CRDS	0.37	0.04	0.66	0.05	−50.80	0.18	−56.1	0.3	17	15	20.2
L4‐08/3/15	CRDS	0.18	0.02	0.32	0.03	−51.74	0.13	−54.3	0.2	12	15	21.9
L5‐09/3/15	CRDS	0.28	0.03	0.50	0.05	−50.64	0.19	−54.6	0.2	19	10	19.9
L6‐11/3/15	CRDS	0.22	0.01	0.40	0.01	−51.31	0.10	−54.5	0.2	3	11	20.7
L1‐12/3/15	CRDS	0.28	0.03	0.52	0.03	−51.16	0.06	−55.3	0.2	12	13	20.8
L6‐13/3/15	CRDS	0.18	0.02	0.31	0.05	−50.63	0.36	−53.1	0.2	15	15	20.7
L3‐22/3/15	CRDS	0.25	0.02	0.45	0.03	−50.50	0.30	−54.5	0.5	6	16	20.4
L4‐23/3/15	CRDS	0.21	0.03	0.39	0.03	−51.29	0.18	−53.2	0.4	9	15	20.5
L6‐26/3/15	CRDS	0.22	0.03	0.43	0.03	−50.28	0.21	−53.7	0.2	−5	16	20.8
L6‐27/3/15	CRDS	0.24	0.01	0.41	0.02	−50.81	0.22	−54.1	0.1	18	10	20.7
L5‐30/3/15	CRDS	0.21	0.03	0.37	0.03	−51.10	0.23	−54.0	0.2	16	12	21.2
L4‐01/4/15	CRDS	0.16	0.03	0.29	0.03	−50.81	0.15	−53.1	0.4	0	12	20.9
L3‐02/4/15	CRDS	0.22	0.02	0.37	0.03	−50.64	0.39	−53.7	0.2	19	15	20.8
L1‐20/4/15	CRDS	0.16	0.03	0.29	0.03	−50.01	0.27	−52.4	0.4	5	13	20.5
L2‐21/4/15	CRDS	0.04	0.06	0.04	0.12	−50.66	0.65	−50.3	0.4	27	9	20.3
L5‐21/4/15	CRDS	0.13	0.04	0.21	0.06	−50.87	0.38	−52.6	0.3	6	13	20.9
L3‐22/4/15	CRDS	0.18	0.02	0.32	0.02	−50.59	0.27	−53.1	0.2	13	15	20.9
L4‐22/4/15	CRDS	0.07	0.02	0.09	0.02	−51.39	0.18	−52.4	0.2	20	14	20.8
L3‐22/4/15	CRDS	0.14	0.03	0.24	0.06	−50.99	0.39	−52.8	0.4	8	13	20.9
L4‐22/4/15	CRDS	0.03	0.02	0.03	0.02	−51.24	0.18	−51.7	0.1	17	14	20.7
**AVG**	**CRDS**	**0.18**		**0.31**		**−51.02**		**−53.4**		**15**		20.7
**STD**		**±0.07**		**±0.13**		**±0.49**		**±1.1**		**±8**		±0.4
**AVG**	**IRMS**	**0.27**		**0.49**		**‐**		**‐**	‐	**8**		20.7
**STD**		**±0.07**		**±0.14**						**±4**		±0.2

**Figure 3:**
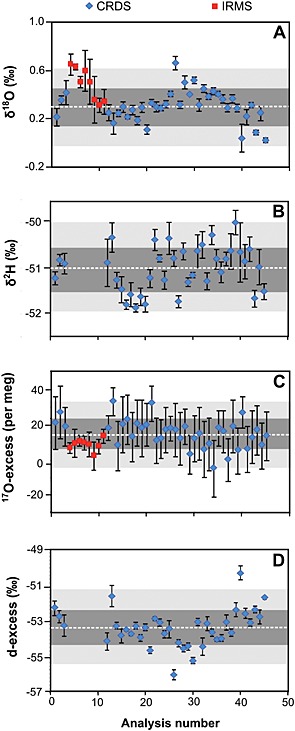
The δ^18^O (A), δ^2^H (B), ^17^O‐excess (C) and d‐excess (D) values of GHW from the repeated analysis of a gypsum standard (NEWGYP), extracted using the WASP and analyzed by CRDS (n = 37) and IRMS (n = 8). δ^17^O displayed a similar trend to δ^18^O. Data are displayed following the order in which the samples were extracted using the WASP. Errors bars refer to the internal error (±1‐sigma obtained from the repeated analysis of the same hydration water (7 injections for CRDS and 3–4 for IRMS). The long‐term means (dashed line) and external errors are shown for ±1‐sigma (deep‐grey shade) and ±2‐sigma (light‐grey shade).

Under correct operating conditions, the long‐term precision of the method was ± 0.07‰ for δ^17^O values, ±0.13‰ for δ^18^O values, and ±0.49‰ for δ^2^H values (n = 37, 7 injections each, ±1SD) for analyses of NEWGYP (δ^17^O = 0.18‰, δ^18^O = 0.31‰ and δ^2^H = −51.02‰) using CRDS (Fig. 3 and Table 2). The IRMS analysis of the same gypsum standard (n = 8, 3 or 4 analyses each) yielded mean values and reproducibility (±1SD) of 0.28 ± 0.07‰ and 0.51 ± 0.14‰ for the δ^17^O and δ^18^O values, respectively, which are within the two‐sigma error of the CRDS measurements (Fig. 3 and Table 2).

No differences in the value of the in‐house water standard were found when the MCM was not used and no systematic drift has been observed in the δ^17^O, δ^18^O and δ^2^H measurements of the standard over time (Fig. 3). In principle, any long‐term drift can be monitored and corrected since a gypsum standard is extracted with each five unknown samples. Because no such drift was observed during the course of this study, there is currently no benefit in correcting the values. However, if a drift is observed in the future, a correction could be applied to improve the long‐term reproducibility of the method.

Gypsum samples with isotopically enriched and depleted hydration water were extracted consecutively in the same line of the WASP in order to determine the potential memory effect of the system. The samples extracted in the first run were found not to influence the δ^17^O, δ^18^O and δ^2^H values of the samples extracted in the second run of the apparatus. The values for the isotopically enriched gypsum (δ^17^O = 13.05‰, δ^18^O = 25.10‰ and δ^2^H = 34.83‰, n = 3, 7 injections each) showed analytical errors (±1SD) of ±0.08‰, ±0.16‰ and ±0.24‰ for the δ^17^O, δ^18^O and δ^2^H values, respectively, and the errors for the isotopically depleted gypsum (δ^17^O = −6.81‰, δ^18^O = −12.86‰ and δ^2^H = −150.39‰, n = 4, 7 injections each) were ±0.06‰, ±0.12‰ and ±0.53‰, respectively (Table 1). These results show that there is no measureable memory effect in the WASP system with our pumping protocol.

For natural gypsum samples, CRDS using the MCM produced values ranging from −1.00‰ to 5.61‰ for δ^17^O, from −1.93‰ to 10.74‰ for δ^18^O and from −53.36‰ to 13.21‰ for δ^2^H (Table 3). The in‐sample reproducibility (±1SD) of the CRDS analyzer for the analysis of natural gypsum samples was typically ±0.03‰ for δ^17^O values, ±0.04‰ for δ^18^O values, and ±0.24‰ for δ^2^H values, found by taking the average of seven consecutive injections for each GHW sample. These results are similar to those observed for the analysis of water standards. Regarding the long‐term reproducibility of natural samples, the replicate GHW extraction and subsequent analysis in different runs of sample Salina 1 (n = 3, 7 injections each) gave an error (±1SD) of ±0.02‰ for δ^17^O values, ±0.03‰ for δ^18^O values, and ±0.16‰ for δ^2^H values (Table 3). This demonstrates the long‐term precision obtainable for the hydration water of natural gypsum samples using our method.

**Table 3:** Isotopic analysis (δ^17^O, δ^18^O and δ^2^H values) of hydration water of a gypsum standard (NEWGYP) and natural samples

**Sample**	CRDS (MCM ON)					CRDS (MCM OFF)					IRMS		
	δ^17^O (‰)	δ^18^O (‰)	δ^2^H (‰)	d‐excess (‰)	^17^O‐excess (per meg)	δ^17^O (‰)	δ^18^O (‰)	δ^2^H (‰)	d‐excess (‰)	^17^O‐excess (per meg)	δ^17^O (‰)	δ^18^O (‰)	^17^O‐excess (per meg)
BG‐10	−1.00 ± 0.02	−1.93 ± 0.02	−53.36 ± 0.21	−37.9 ± 0.2	**20 ± 13**	−0.89 ± 0.01	−1.78 ± 0.03	−52.90 ± 0.14	−38.6 ± 0.2	**57 ± 11**	−1.01 ± 0.02	−1.96 ± 0.04	**25** ± **7**
CRI‐01	−0.06 ± 0.02	−0.15 ± 0.04	−41.91 ± 0.23	−40.7 ± 0.2	**15 ± 7**	−0.04 ± 0.02	−0.16 ± 0.04	−42.3 ± 0.30	−42.9 ± 0.3	**54 ± 12**	0.09 ± 0.01	0.12 ± 0.01	**27** ± **5**
NEWGYP (n=36/8)*	0.17 ± 0.07	0.30 ± 0.13	−51.03 ± 0.48	−53.4 ± 1.0	**15 ± 8**	0.12 ± 0.04	0.15 ± 0.05	−51.23 ± 0.35	−53.0 ± 0.6	**42 ± 12**	0.27 ± 0.07	0.49 ± 0.14	**8** ± **4**
SBL‐2.3	1.55 ± 0.01	2.92 ± 0.03	−27.71 ± 0.27	−51.6 ± 0.3	**−7 ± 15**	1.88 ± 0.02	3.40 ± 0.02	−27.47 ± 0.11	−54.7 ± 0.1	**77 ± 13**	1.85 ± 0.01	3.52 ± 0.02	**−7** ± **1**
SBL‐8	1.68 ± 0.02	3.13 ± 0.02	−27.72 ± 0.07	−53.2 ± 0.1	**7 ± 15**	1.74 ± 0.02	3.17 ± 0.02	−28.31 ± 0.07	−53.7 ± 0.2	**61 ± 14**	1.90 ± 0.01	3.60 ± 0.03	**1** ± **5**
PI 6A‐13H‐2 7–8 cm	4.46 ± 0.01	8.53 ± 0.02	3.76 ± 0.14	−64.5 ± 0.2	**−26 ± 12**	5.08 ± 0.04	9.41 ± 0.05	6.93 ± 0.20	−68.4 ± 0.4	**123 ± 18**	4.87 ± 0.00	9.30 ± 0.01	**−30** ± **2**
PI 6C‐7H‐2 25–26 cm	4.89 ± 0.03	9.30 ± 0.05	9.76 ± 0.26	−64.7 ± 0.3	**−17 ± 9**	5.32 ± 0.02	9.74 ± 0.02	11.21 ± 0.06	−66.8 ± 0.2	**190 ± 7**	4.98 ± 0.02	9.51 ± 0.03	**−30** ± **2**
Salina 1 (n=3)*	5.61 ± 0.02	10.74 ± 0.03	13.21 ± 0.16	−72.7 ± 0.2	**−48 ± 8**	5.74 ± 0.04	10.74 ± 0.04	13.55 ± 0.18	−72.4 ± 0.4	**72 ± 16**	5.88 ± 0.03	11.26 ± 0.07	**−49** ± 5

* Samples analyzed in replicate (in these cases, the analytical error refers to the external precision, taking the average of the indicated number of samples).

**Figure 4:**
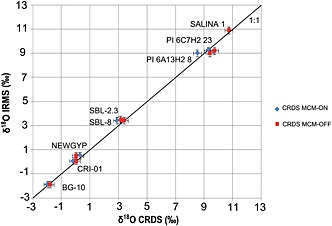
Cross‐plot of δ^18^O values in GHW of natural samples analyzed by CRDS/MCM compared with measurements of the same samples by IRMS. The long‐term ±2‐sigma error of the method (0.26‰ for the δ^18^O values from the CRDS measurements and 0.28‰ for the δ^18^O values from the IRMS analyses) is given for all samples. The in‐sample ±2‐sigma errors (7 injections for CRDS and 3–4 for IRMS) are smaller than the data symbols.

For most natural samples, the δ^17^O, δ^18^O and δ^2^H values of GHW did not differ significantly when the MCM was used or not (within the two‐sigma error) from the results obtained by IRMS (Fig. 4). However, the ^17^O‐excess differed considerably between CRDS and IRMS when the MCM was not used (see next section). The ^17^O‐excess values measured by CRDS were considerably higher than those obtained by IRMS when the MCM was turned off. The difference in ^17^O‐excess values with MCM On and Off reflects the degree of spectral interference caused by contamination.

### ^17^O‐excess and deuterium‐excess in GHW

**Figure 5:**
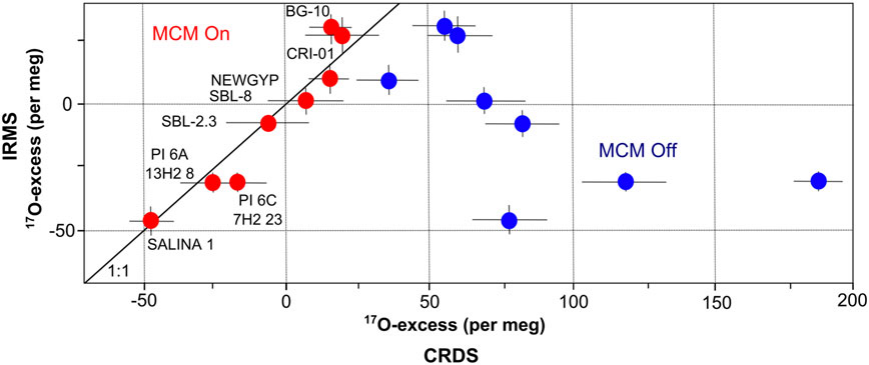
Cross‐plot of ^17^O‐excess in synthetic and natural gypsum analyzed by CRDS and IRMS. The analyses by CRDS that used the MCM yielded results similar to those obtained by IRMS. Error bars refer to the consecutive analyses (7 injections for CRDS and 3–4 analyses for IRMS) of the same hydration water.

CRDS and IRMS produced similar ^17^O‐excess values for the NEWGYP standard (15±8 per meg for CRDS and 8±4 per meg for IRMS) when the MCM accessory was used with the CRDS analyzer (Table 3 and Fig. 5). However, the ^17^O‐excess value differed considerably (~25 per meg higher for CRDS) when the MCM was configured in WARM mode (Fig. 5).

Similarly, the ^17^O‐excess values in natural samples analyzed by CRDS and IRMS did not agree when the MCM was not used (Fig. 5 and Table 3). The ^17^O‐excess difference was up to ~40 per meg in the GHW from the hydrothermal gypsum sample (CRI‐01 and BG‐10), and the results from lake samples showed an even larger disagreement of up to ~200 per meg in GHW (e.g. PI 6C‐7H‐2 26 cm). For all samples, the ^17^O‐excess of hydration water was greater when the MCM was not powered on. By contrast, the ^17^O‐excess results from CRDS were similar to those achieved by IRMS when the MCM was used (Table 3), resulting in a close 1:1 relationship (Fig. 5).

In previous work on the isotopic composition of GHW in lake samples measured by CRDS, Hodell *et al*.^6^ analyzed their spectra using Picarro's ChemCorrect software that identifies irregularities caused by traces of hydrocarbons.^27^ These authors found that none of their samples showed any signs of spectroscopic interference affecting the δ^18^O or δ^2^H values, probably because the hydrocarbons and fatty acids present in the gypsum tend to be of longer mean chain length, as suggested in earlier work.^28^ In our study, we analyzed some similar lake gypsum samples, which showed the largest discrepancies when the MCM was not used (PI 6A‐13H‐2 8 cm, PI 6C‐7H‐2 26 cm). Our results strongly indicate that contaminant gases released from natural gypsum samples (VOCs, H_2_S, etc.) during dehydration cause spectral interferences at the wavenumber used to determine ^1^H_2_^17^O by CRDS (7193 cm^−1^),^16^ but not in a meaningful way to the ^1^H_2_^18^O and ^2^H^1^H^16^O signals; thus, although the ^17^O‐excess is affected by these contaminants, the δ^18^O and δ^2^H values are not.

Use of the micro‐combustion module (MCM) removes impurities that affect the ^17^O‐excess determination. As stated above, the differences in ^17^O‐excess in natural samples in our MCM On/Off experiments are greater in the case of gypsum samples generated in organic‐ and microbe‐rich environments (e.g. lake sediments). This, as expected, points to higher concentration of contaminants in these types of material and suggests that the cause of the spectral interferences could be H_2_S or organic compounds released by natural gypsum samples during dehydration. Further evidence for the presence of impurities in GHW is given by a strong smell of H_2_S in water after extraction, especially in water from lacustrine gypsum samples.

Our results reveal that the use of the MCM accessory is crucial for accurate determination of the ^17^O‐excess in GHW by CRDS. In earlier work, this device was found to be necessary for isotopic analysis of water samples with high concentrations of organic compounds, such as water extracted from the xylem of plants that usually contains alcohols that spectroscopically interfere with the CRDS analysis.^18^, ^19^ To date, the efficiency of the MCM has been demonstrated for the removal of alcohols and other organic compounds from water.^19^ However, catalytic oxidation of other substances also occurs, such as H_2_S, VOCs and long‐chain compounds, and this is important for the analysis of GHW samples. Although this accessory removes most impurities, we suspect that organic‐rich gypsum samples (e.g. those from lake sediments) may require additional pre‐treatment to remove organic compounds (H_2_O_2_, sodium hypochlorite, etc.). However, any pre‐treatment procedure will require testing to ensure that it does not alter the δ^18^O and δ^2^H values of the hydration water.

Using the MCM, the long‐term precision (±1SD) of our method for ^17^O‐excess determination by CRDS of the analytical‐grade gypsum standard (NEWGYP) was ±8 per meg (n = 37) (Table 2). In addition, no systematic drift in the ^17^O‐excess of the standard has been observed with time (Fig. 3). Remarkably, this reproducibility is better than the typical in‐sample precision obtained from seven consecutive injections (±13 per meg) observed in both water standards and GHW. Similar precision (typically ±15 per meg) is also obtained by randomly choosing the ^17^O‐excess of injections from NEWGYP samples, extracted on different days and analyzed in different CRDS analyzer runs. This is a consequence of the minimized importance of drift, memory effect and potential isotopic fractionation occurring during the ^17^O‐excess measurements, compared with the measurement of the δ^17^O and δ^18^O values. As demonstrated by Barkan and Luz,^17^ and later by Schoenemann *et al*.,^26^ the errors in the δ^17^O and δ^18^O values are covarying. This means that any isotopic fractionation during the analytical procedure may affect the individual δ^17^O and δ^18^O values, but does not change significantly the relative difference between the δ^17^O and δ^18^O values (^17^O‐excess).

Likewise, the repeated extraction and measurement (n = 3, 7 injections each) of one natural gypsum sample (Salina 1) by CRDS using the MCM produced an analytical error (±1SD) of ±7 per meg. This indicates that the long‐term ^17^O‐excess precision of our method using CRDS is similar for synthetic and natural gypsum when the MCM is powered on. The long‐term precision is also similar to that obtained for the analysis of water standards, as observed from the repeated measurement of our internal water standard (SPIT, ±8 per meg, n = 23) in different runs over the period of this study. No measurable memory effects were detected in our experiments with ENR‐gyp and DPL‐gyp for the ^17^O‐excess (Table 1).

The repeated extraction of our NEWGYP standard (n = 8) and subsequent analysis by IRMS produced a typical in‐sample precision of ±6 per meg (3 or 4 consecutive analyses of each hydration water sample) and an external precision of ±4 per meg, which is similar to the typical precision achieved for water standards using CoF_3_ fluorination IRMS^17^, ^26^ and better than the precision obtained by CRDS (±8 per meg).

A distinct advantage of CRDS over IRMS is the possibility of simultaneously determining ^2^H/^1^H, along with ^17^O/^16^O and ^18^O/^16^O, on the same hydration water sample. This enables the calculation of both d‐excess and ^17^O‐excess in GHW. The long‐term d‐excess of the NEWGYP standard (n = 37) determined using CRDS was −53.4 ± 1.1‰ (Fig. 3 and Table 2). This reproducibility is comparable with the long‐term precision observed from the repeated measurement of our SPIT water standard (±0.9‰, n = 23). No systematic drift in the d‐excess of the NEWGYP standard has been observed with time (Fig. 4). As for the natural gypsum samples, the d‐excess in GHW analyzed by CRDS using the MCM ranged from −39.7‰ to −71.9‰. As expected, the values are positively correlated with those of the ^17^O‐excess across the dataset.^11^

### Distillation of brines and isotopic analysis by CRDS

The isotopic values of the untreated and distilled standards agree within the internal error of the CRDS water analyzer (Table 4), suggesting that there was complete water vapour recovery in the cryogenic traps and no isotopic fractionation during distillation in the WASP. The reproducibility (±1SD) for two repeated analyses (7 injections each) of the distilled brines was better than ±0.04‰ and ±0.06‰ for the δ^17^O and δ^18^O values, respectively, and better than ±0.27‰ for the δ^2^H values (Table 4), which is similar to the long‐term precision of the CRDS analyzer for the analysis of water standards. When comparing the untreated and the distilled standards, the derived d‐excess and ^17^O‐excess values differed by less than 0.3‰ and 10 per meg, respectively. This demonstrates that the WASP can be used for the distillation of saline solutions for isotopic analysis by CRDS, with no contamination of the analyzer by salt deposition. However, earlier investigations found that isotopic fractionation may occur during distillation of highly saline brines.^29^ The degree of fractionation depends on the nature and the molar concentration of salts, and can be corrected using 'salt effect' coefficients as long as the activity of the major elements (Ca^2+^, Mg^2+^, K^+^) is known (i.e.^30^). Additional corrections may need to be applied to obtain accurate isotopic values after distillation and CRDS analysis of brines.

**Table 4:** Isotopic composition of water samples distilled using the WASP and analysed by CRDS

Sample	δ^17^O (‰)	1σ (‰)	δ^18^O (‰)	1σ (‰)	δ^2^H (‰)	1σ (‰)	d‐excess (‰)	1σ (‰)	^17^O‐excess (per meg)	1σ (per meg)
JRW	−9.99	0.01	−18.85	0.02	−146.01	0.19	**5.6**	**0.2**	**−4**	**13**
JRW‐WASP	−10.02	0.03	−18.90	0.05	−146.19	0.20	**5.9**	**0.2**	**2**	**13**
BOTTY	−3.95	0.02	−7.52	0.02	−50.18	0.21	**10.6**	**0.2**	**20**	**13**
BOTTY‐WASP	−3.96	0.02	−7.54	0.03	−50.22	0.11	**10.7**	**0.3**	**24**	**10**
SPIT	−0.07	0.02	−0.13	0.03	0.16	0.14	**1.5**	**0.3**	**−6**	**11**
SPIT‐WASP	−0.08	0.02	−0.17	0.02	−0.17	0.20	**1.4**	**0.3**	**6**	**13**
ENR	5.64	0.02	10.76	0.01	40.23	0.10	**−46.4**	**0.1**	**−36**	**18**
ENR‐WASP	5.55	0.02	10.59	0.02	39.58	0.12	**−45.6**	**0.1**	**−37**	**12**
DEPO‐03A	3.03	0.03	5.81	0.02	21.34	0.16	**−25.3**	**0.2**	**−43**	**21**
DEPO‐03B	3.03	0.02	5.80	0.02	21.33	0.08	**−25.3**	**0.2**	**−40**	**17**
DEPO‐06A	4.15	0.01	7.95	0.01	34.02	0.18	**−29.9**	**0.2**	**−47**	**10**
DEPO‐06B	4.20	0.03	8.03	0.03	34.40	0.11	**−30.1**	**0.2**	**−36**	**16**

## Conclusions

The method described enables the determination of the isotopic composition (δ^17^O, δ^18^O, and δ^2^H values) of gypsum hydration water (GHW) using the newest generation of CRDS analyzer. Our procedure presents several analytical advantages over earlier methods, including better long‐term precision, higher sample throughput, reduced sample size and less memory effect between consecutive samples.

Simultaneous ^17^O‐excess and d‐excess values can be obtained by this method in GHW and brines. This can provide additional information about the conditions under which the gypsum formed and subsequently interacted with other fluids after deposition. Although the present procedure has been initially tested for the analysis of gypsum, other hydrated minerals could also be extracted. This opens up a broad field of possible future applications using this technique.

## Supporting information

Supporting info item
