# Supplementary material for: Simultaneous analysis of 17O/16O, 18O/16O and 2H/1H of gypsum hydration water by cavity ring‐down laser spectroscopy
Source: Rapid Commun Mass Spectrom. 2015 Sep 27;29(21):1997–2006. doi: 10.1002/rcm.7312 (PMC5132057; doi:10.1002/rcm.7312)
Supplement: Supplementary file 1 — Supporting info item [file RCM-29-1997-s001.pdf]

Supplementary information

Simultaneous analysis of $^{17}O/^{16}O$, $^{18}O/^{16}O$ and $^{2}H/^{1}H$ of gypsum hydration water by cavity ringdown laser spectroscopy

Fernando Gázquez[1*], Ian Mather[1], James Rolfe[1], Nicholas P. Evans[1], Daniel Herwartz[2], Michael Staubwasser[2] and David A. Hodell[1]

[1]Godwin Laboratory for Palaeoclimate Research. Department of Earth Sciences. University of Cambridge. Downing Street, Cambridge, Cambridgeshire, CB2 3EQ, United Kingdom

[2]Institute für Geology und Mineralogy. Universität zu Köln. Greinstrasse. 4-6, 50939, Köln, Germany

*corresponding author (f.gazquez@ual.es)

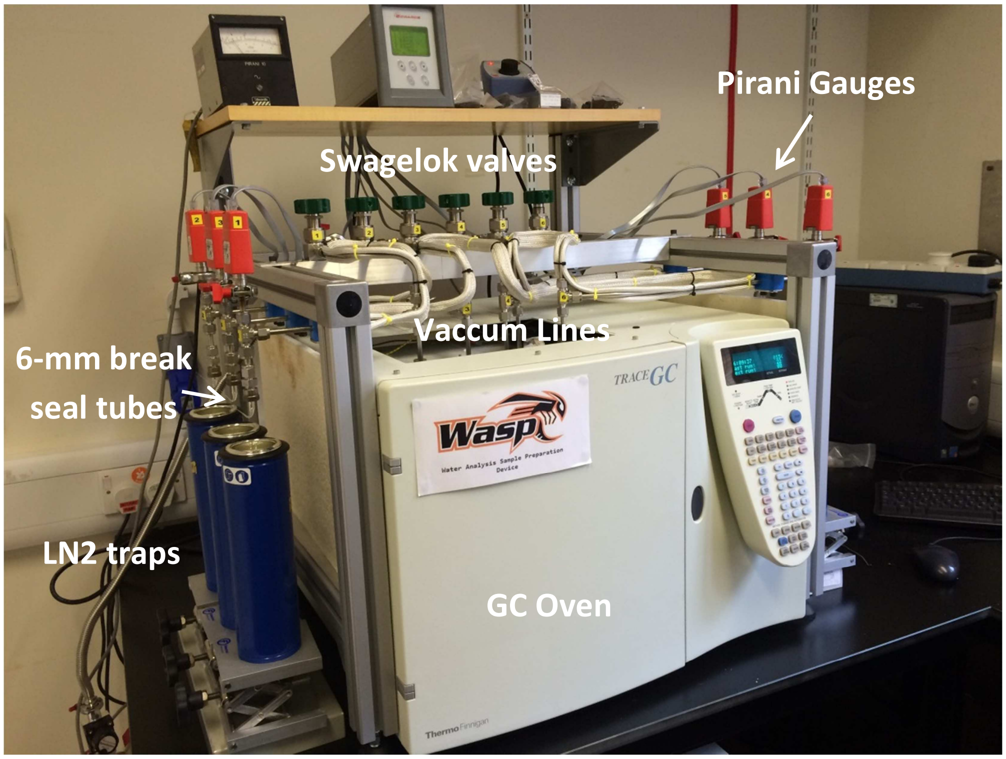

Figure S1. Frontal view of the Water Analyzer Sample Preparation (WASP) apparatus.

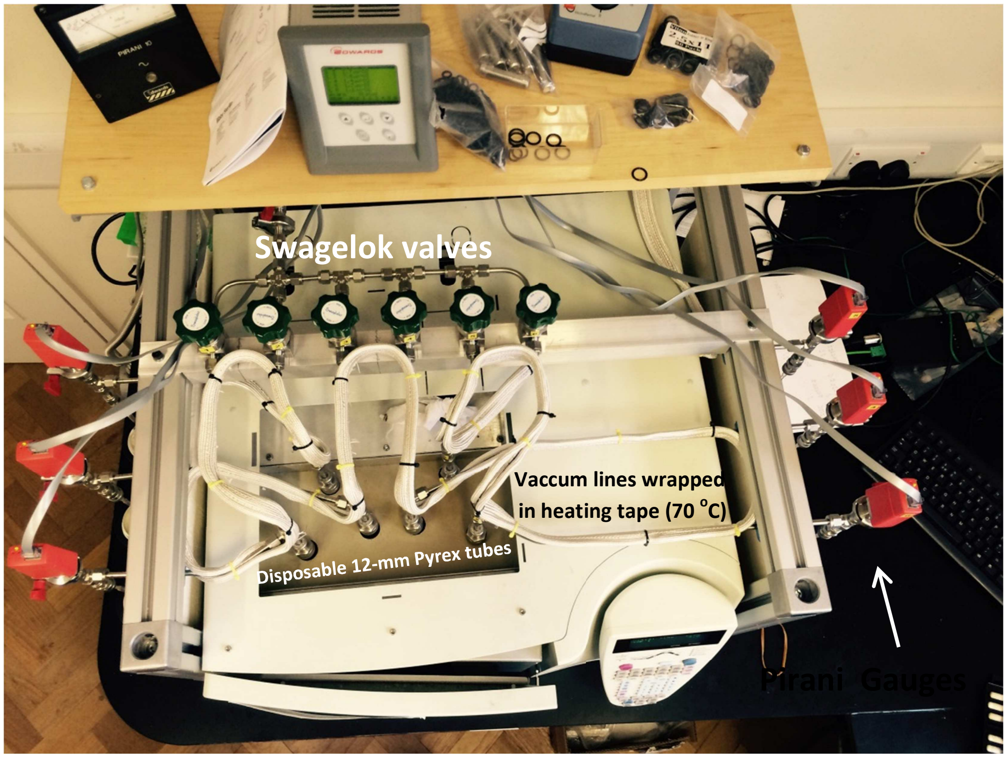

Figure S2. Bird's-eye view of the WASP apparatus.

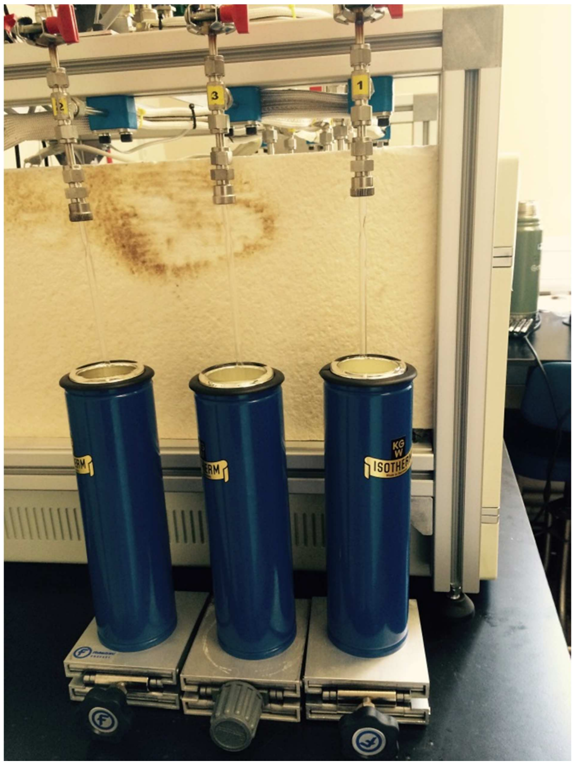

Figure S3. 6-mm break-seal tubes and LN2 traps.

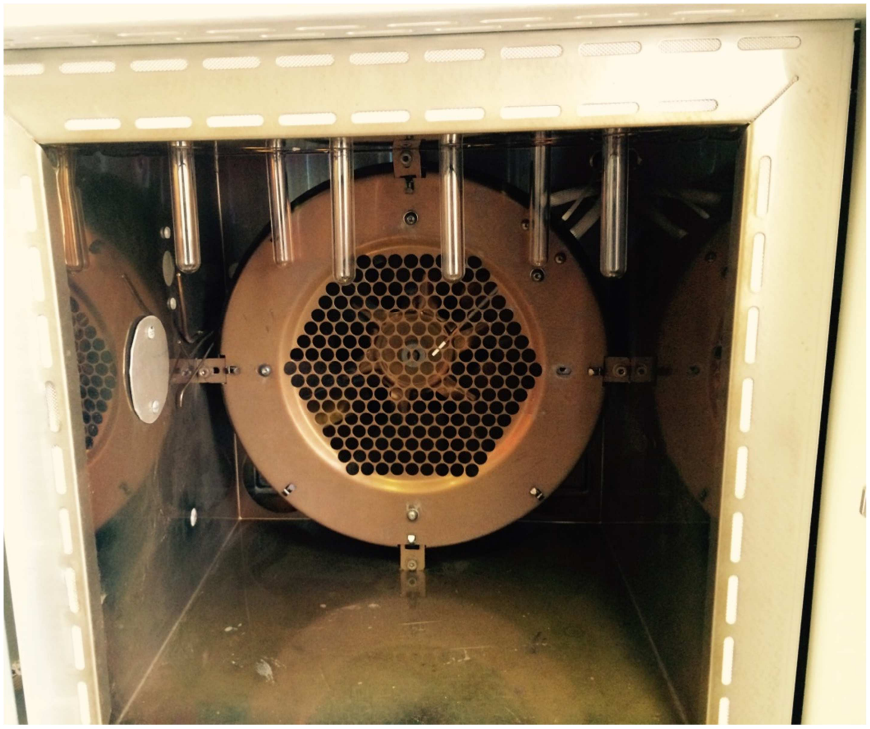

Figure S4. Internal view of the GC oven. Note the 6 disposable 12-mm Pyrex tubes for gypsum sample loading.

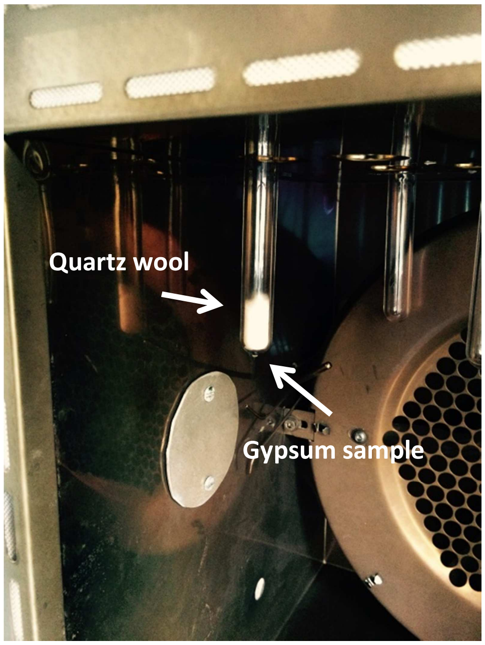

Figure S5. 12-mm Pyrex tubes loaded with a gypsum sample and topped with 3 cm of quartz wool before hydration water extraction.

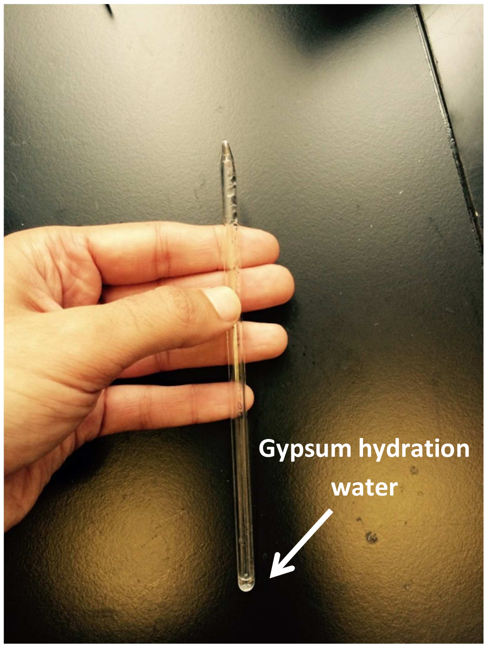

Figure S6. Gypsum hydration water recovered after a WASP run in a 6-mm break-seal tube.
